# DengueFog: A Fog Computing-Enabled Weighted Random Forest-Based Smart Health Monitoring System for Automatic Dengue Prediction

**DOI:** 10.3390/diagnostics14060624

**Published:** 2024-03-15

**Authors:** Ashima Kukkar, Yugal Kumar, Jasminder Kaur Sandhu, Manjit Kaur, Tarandeep Singh Walia, Mohammed Amoon

**Affiliations:** 1 Chitkara University Institute of Engineering and Technology, Chitkara University, Punjab 140401, India; ashima@chitkara.edu.in; 2 Department of Computer Science and Engineering, Jaypee University of Information Technology, Waknaghat, Solan 173234, India; yugal.kumar@juit.ac.in; 3 Department of Computer Science & Engineering, Chandigarh University, Gharuan, Mohali 140413, India; 4 School of Computer Science and Artificial Intelligence, SR University, Warangal 506371, India; man-jit@ieee.org; 5 School of Computer Application, Lovely Professional University, Phagwara 144411, India; taran_walia2k@yahoo.com; 6 Department of Computer Science, Community College, King Saud University, P.O. Box 28095, Riyadh 11437, Saudi Arabia

**Keywords:** dengue, fog computing, cloud computing, IoT, random forest

## Abstract

Dengue is a distinctive and fatal infectious disease that spreads through female mosquitoes called Aedes aegypti. It is a notable concern for developing countries due to its low diagnosis rate. Dengue has the most astounding mortality level as compared to other diseases due to tremendous platelet depletion. Hence, it can be categorized as a life-threatening fever as compared to the same class of fevers. Additionally, it has been shown that dengue fever shares many of the same symptoms as other flu-based fevers. On the other hand, the research community is closely monitoring the popular research fields related to IoT, fog, and cloud computing for the diagnosis and prediction of diseases. IoT, fog, and cloud-based technologies are used for constructing a number of health care systems. Accordingly, in this study, a DengueFog monitoring system was created based on fog computing for prediction and detection of dengue sickness. Additionally, the proposed DengueFog system includes a weighted random forest (WRF) classifier to monitor and predict the dengue infection. The proposed system’s efficacy was evaluated using data on dengue infection. This dataset was gathered between 2016 and 2018 from several hospitals in the Delhi-NCR region. The accuracy, F-value, recall, precision, error rate, and specificity metrics were used to assess the simulation results of the suggested monitoring system. It was demonstrated that the proposed DengueFog monitoring system with WRF outperforms the traditional classifiers.

## 1. Introduction

The illnesses transmitted by mosquitoes are deadly in nature and spread swiftly from infected to uninfected individuals via bacteria, viruses, and parasites [1]. The bite of a female mosquito infected with the virus is the primary cause of transmission [2]. In addition, the infected individual needs to be monitored regularly to diagnose a particular disease and to determine a suitable legal therapy [3] These lethal infections include filariasis, malaria, West Nile fever, chikungunya, Zika virus, yellow fever, and dengue fever. The speedy blowout of this pollution is an outcome of a developing transportation network, environmental and climatic change, and the inability to control mosquito reproduction [4]. The cautioning symptoms and indicators of these lethal diseases remain almost identical; hence, it is quite difficult to distinguish and classify the exact condition. Therefore, for identifying the specific condition, patients must undertake numerous medical tests [5]. Because of the growing number of infected individuals and insufficient health care resources, these tests are not administered to the majority of patients. Consequently, subsequent treatment and imprecise diagnosis contribute to a high fatality rate, thereby promoting a hike in mosquito-borne diseases [6]. Thus, it is a big problem for government health care insurance companies to identify mosquito-borne illnesses at an initial phase and prevent their rapid transmission. To treat the majority of mosquito-borne diseases, there is no specific therapy or drug [7]. Thus, an intelligent framework is required to identify and prevent the rapid spread of mosquito-borne illnesses from the outset. The patients affected by a disease transmitted by mosquitoes require regular surveillance. Patients are not always able to visit a hospital or health care facility for routine checkups. Thus, a remote health care monitoring system may be created to promote ubiquitous health care services utilizing emerging technical interventions such as Internet of Things (IoT), wearable devices, cloud computing, wireless sensors, fog computing [8,9]. These technologies can be utilized for the surveillance of key patient indicators and the provision of compassionate treatment. In modern sensing technologies, numerous wearable gadgets such as inconspicuous, smart fabrics, and printable electronic tattoos are employed [10,11,12,13]. The purpose of these gadgets is to collect individual health data in order to anticipate a healthy lifestyle [14,15]. In addition, wireless sensor mobile computing paradigms are frequently utilized in the health care arena for data collecting and processing, despite the fact that the storage capacity of mobile phones is ample for processing health-related data [16,17].

Due to centralized storage and advanced calculation facilities [18,19], cloud computing is also utilized in medical informatics. Numerous health care applications employ IoT as the vital acquisition module for creating a smart environment [20,21]. IoT is capable of managing, storing, and analyzing voluminous amounts of data. Cloud computing, on the other hand, offers resources based on the pay-per-service model. Multiple IoT-based applications make use of these services. One of them is the remote health care monitoring system [22,23,24]. Various computer tools, such as cloud computing, wireless sensors, and mobile computing, are being used to boost the quality of health care amenities [25]. The advantages of cloud computing include storage size, availability, cost-effectiveness, scalability, and accessibility. These characteristics help government agencies create remote health monitoring systems [26]. In addition, unprecedented volumes of health data are kept in the cloud-based data centers. Consequently, the price of health care amenities is drastically decreased [27]. Moreover, the framework allowed by cloud computing efficiently monitors patients affected by mosquito-borne diseases and seamlessly uses the medical records across clinics for efficient administration of health statistics [28,29]. Managing enormous amounts of data on the cloud, however, is extraordinarily difficult, and it slows the transmission across the internet, which can have dire effects such as endangering the lives of patients [30]. In addition, processing overhead, traffic across network, movement, location consciousness, and correspondence overhead may develop. In context of patients’ personal information in medical informatics [31,32,33], privacy breaches and threats are another concern. Fog computing might be seen as a solution to the aforementioned issues and limitations of cloud computing. It can function as an intermediate between the end user and the cloud server for providing health care services and resources [34].

Between centralized cloud infrastructure and the IoT is a stratum of fog computing for managing messaging overhead, latency, decision-making, resident storage, and information preprocessing problems between the end user and the cloud server [35,36,37]. Integration of IoT, fog computation, and the cloud enhances scalability and agility for controlling the mosquito-borne diseases by incorporating topographical areas and assessments based on real-world data analytics [38]. It provides enhanced support to the Nano Data Centers in terms of data storage and consumes minimum power compared to cloud computing. It is the consequence of time consumption, application type, download counts, information pre-loading, upgrades, and type of network accessed [39].

In recent years, researchers have paid a great deal of attention to eHealth care applications in an effort to attain a healthy lifestyle with confidence and excitement, as well as to improve well-being. The e-health applications must be accurate and must focus on the patient. Such applications need constant patient health statistics, yet those data are insufficient for accurately forecasting the illness status. It further needs contextual data for diagnostic extraction [40]. Therefore, contextual information, patient input, and patient profile information may be used to generate more precise patient-centric suggestions and diagnoses [41]. The projected fog-based health monitoring system has the following prime objectives:

▪ To develop a fog and IoT- based health monitoring arrangement to allow remote diagnosis of dengue infection based on a patient’s health symptoms.
▪ To provide immediate treatment to dengue-infected patients, monitor infected patients, and routinely issue health-specific alert messages. Continuous monitoring and timely notifications of blood pressure fluctuations should also be provided, allowing users and physicians to make health decisions.
▪ To have an effective framework for sharing medical records in order to give preventive measures and recommendations based on the present condition of hypertension.

## 2. Related Work

In order to offer a diagnosis, Shah et al. [42] integrated and analyzed historical patient data with real-time patient data. The authors also looked into the problems with service quality for medical applications. Cloud to fog (C2F) and IoT computing have been used to design the u-health care monitoring system by Nandyala et al. [43]. Through end points, the proposed system increases communication between hospitals and smart homes. It has been found that the suggested system meets all the needs of evolving models and offers quick processing with fewer delays than cloud-based systems. A trustworthy and adaptable health care monitoring system for disease diagnosis was created by Costanzo et al. [44]. Wearable technology and embedded technologies are discussed in the suggested system. The main goal is to use mobile devices to monitor patients who are stationed far away. For quick patient rescue in an emergency, the suggested monitoring technique is used for interfacing by means of the first-aid software.

Oluwagbemi et al. [45] used fuzzy logic and expert systems to construct their Ebola fuzzy informatics system. The suggested approach was made to diagnose and suggest treatment for the Ebola virus disease. A health care system was introduced by Sood et al. [46] to track and distinguish between the numerous diseases spread by mosquitoes. IoT sensors, fog, and cloud computing make up the main components of the system. The suggested system’s goal is to regulate diseases at their earliest stages. The suggested structure calculates similarity factors to distinguish between diseases. A security-based architecture for geographically distant health care systems was created by Thota et al. [47]. The suggested architecture enables asynchronous communication between cloud-based health applications and data servers. The tracking, identification, and security of authorization and authentication for all devices are the primary goals of the proposed design. For a fog-based eHealth architecture, Venckauskas et al. [48] offered a protected self-authenticable transfer protocol. The communication between the fog nodes and the edge nodes is supplied by the suggested protocol. Datagram Transport Layer Security (DTLS) and User Datagram Protocol are replaced with the suggested protocol as a secure transport for Constrained Application Protocol (CoAP) (UDP). A health care system [49] was used to manage mosquito infections at an initial stage. The suggested solution uses wearables and IoT devices to gather patient data. An account of the numerous Ayurvedic, complementary, and contemporary homeopathic treatments for the ZIKV virus was presented by Saxena et al. [50]. Additionally, the author discussed potential treatments for ZIKV infection.

According to Ginier et al. [51], Zika fever might be mistaken for dengue fever, though Zika infection seldom causes fever. It has been observed that the only symptoms of a Zika infection are skin rashes and slight edema in the patient. Reverse Transcription—Polymerase Chain Reaction (RT-PCR) assay was used by Pabbaraju et al. [52] to identify the Zika, Dengue, and Chikungunya viruses. In order to identify and distinguish between these viruses for the purpose of proper therapy, the RT-PCR assay was used for testing the blood of a patient. A web interface was created by Campion et al. [53] to display information regarding the frequency of the West Nile virus, the density of mosquitoes, and the weather. Google Maps was used in the proposed interface. An age tracking tool was developed by Lambert et al. [54] to accurately predict the age of the mosquito. The suggested method made use of boosted regression trees, random forests, main components regression, and neural networks with near-infrared spectroscopy, among other machine learning approaches. The authors also made the argument that a crucial parameter for killing adult mosquitoes is the mosquito’s age. This objective age assessment generates a precise mosquito population.

Kirk et al. [55] created the DEAR (Detect, Evaluate, Assess, and Recommend action) decision-making system. This system’s primary objectives are to identify environmental changes, make risk assessments, and provide real-time advice for mitigating mosquito illness outbreaks. A health care system was put into place by Devarajan et al. [56] dealing with the Parkinson’s disease. The suggested system examined patient voice samples to suggest the best course of action. In the suggested architecture, fog computing serves as a midway layer in the end user and the cloud server. Further, the classification of Parkinson and non-Parkinson subjects was performed using the fuzzy k-nearest neighbor (FKN) classifier, case-based reasoning (CBR) classifier. A health monitoring system utilizing cloud concept, multiple machine learning methods, and IoT structure was described by Kaur et al. [57]. The recommendations for diagnostics were provided based on the past data stored in the cloud. The judgments of how to hide the numerous patterns in the database were also aided by the suggested method. Additionally, the authors used the accuracy parameter to provide comparative analysis of prediction model’s performance. Parthasarathy et al. [58] proposed LMM system for joint inflammatory disease made use of wearable sensor devices and uric acid sensors as a component of IoT infrastructure. The suggested technique is also utilized to transform health information and identify foot motion in order to diagnose GOUT arthritis.

A novel model called HealthFog was created by Tuli et al. [59] for the automatic analysis of cardiac disorders. HealthFog integrates edge computing (EC) hardware with deep learning (DL). Additionally, the suggested model offered fog services via IoT devices and maintained medical data in accordance with user requests. Using FogBus, the implementation time, latency, power consumed, accuracy, bandwidth of network, and jitter of HealthFog were evaluated. The findings demonstrated that HealthFog offers the highest level of service quality and forecast accuracy. Priyadarshini et al. [60] developed the DeepFog health care model to forecast overall wellness. Fog computing and deep learning were combined to create DeepFog. It used fog computing to gather patient data and deep neural networks to forecast three aspects of well-being, including stress level, hypertension attacks, and diabetes. The recommender system was established by Jabeen et al. [61] to diagnose heart illness. The primary purpose of the suggested system is providing consumers nutrition and exercise advice. There are four sections to the suggested scheme. The patient’s data are gathered in the first section utilizing biosensors, and then they are sent to the server via an IoT environment.

A cyber–physical localization (CPL) system was proposed by Sood et al. [62] constructed on the concepts of cloud computation and neuro-fuzzy implication. The fundamental goal of the projected system is to assess the jeopardy of coronary heart disease, for tracking patients’ ECG readings, to inform users and specialists when readings are aberrant, and to suggest medications and preventive measures in accordance with risk category. To alleviate the lack of domain expertise between computer scientists and physicians, Gu et al. [63] established a Diagnostic Knowledge Model (DKM) for classifying the clinical conditions. The suggested system’s main goals are to discharge the health staff of the hefty weight of hospital duties and to offer appropriate decision-support. The proposed system incorporated medical devices and made use of knowledge systems using the Component-Based Medical Cyber–Physical System framework (CBMCPS).

A health care system was introduced by Sood et al. [64] to identify early-stage hypertension individuals based on user health data. The suggested method continuously evaluates and keeps track of the patients’ blood pleasure. Four phases make up the planned system. IoT sensors placed at the fog layer are used at the initial stage to gather user data. Artificial neural networks are used in the second phase to forecast the likelihood of an attack of hypertension. A health care system was developed by Lakshmanaprabu et al. [65] employing IoT structure, MapReduce, the Enhanced Dragonfly Algorithm, and RF classifier. There are two phases to the suggested system. Patients’ data are gathered in the first phase utilizing IoT devices and the MapReduce method. During the second stage, the dataset’s properties are chosen using an upgraded version of the Dragonfly algorithm. The final phase uses an RF classifier to categorize the various diseases according to chosen criteria.

A hybrid framework was created by Anand et al. [66] to categorize the hepatic syndrome. The medical information was first categorized according to the presence of diseases. The updated Particle Swarm Optimization technique was created in second stage to separate the attributes from the health dataset. The updated artificial neural network was used to categorize diseases in third step. Sood et al. [67] suggested a diagnostic system with NB network and fog computing for infection detecting. The planned system also incorporates Social Network Analysis (SAS) in the cloud subsystem to offer a GPS-based worldwide risk assessment of dengue infection on Google Maps and for preventing the spread of the infection. Sood et al. [68] developed an IoT-based fog-cloud diagnosed system for controlling and detecting dengue infection in 2021. The SVM methods is utilized in the fog layer for evaluation. In addition, the proposed system uses Google Maps and Temporal Network Analysis (TNA) to classify places as infected, uninfected, or risky. Suggala et al. [69] introduced a novel dengue prediction method using fog computing. The dengue infected was detected by checking the similarity factors between the disease and the users.

Comparative analysis of the aforementioned literature is provided in Table 1.

## 3. Proposed Monitoring System Based on Fog Computing

**Figure 1:**
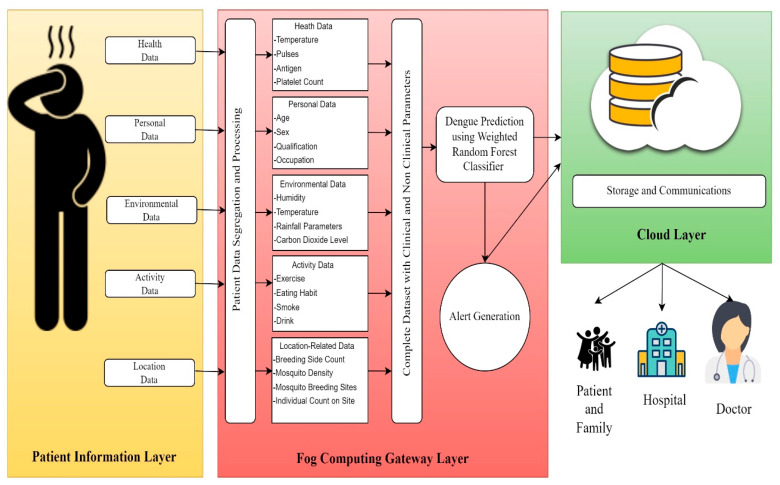
Architecture of proposed fog computing-based monitoring system.

This section exemplifies the discussed DengueFog system. The main aim of DengueFog system is early prediction or identification, prevention, and monitoring of dengue infection. Figure 1 illustrates the architecture of the proposed fog-based smart health monitoring system. The DengueFog system can be utilized to predict the dengue infection as well as inform the concerned stakeholders by generating the alarm in the condition of a positive result. The DengueFog system has two spaces, namely cyberspace and the physical space. The breeding places, mosquito count, patient’s personal information, symptoms, and contact information are all captured in the physical space. In cyberspace, fog and cloud computing are combined to process the data and a powerful cyber–physical system is designed for health care application areas. Cloud servers are used to store vast amounts of data and process large amounts of data. However, an intermediary fog computing system was deployed to deliver lower latency and location consciousness. It also allows real-time applications to use emergency notification services. A smart health monitoring system consists of four layers. The layers are data collection layer (DCL), fog computing gateway layer (FCGL), cloud processing layer (CPL), and end user layer (EUL). The DCL collects the real-time information from diverse sensors, including the physiological data of users, mosquito density, geographical location, and contextual information. This information or data is represented as environment data, health data, behavioral data, location data, motion data, and private data.

**Figure 2:**
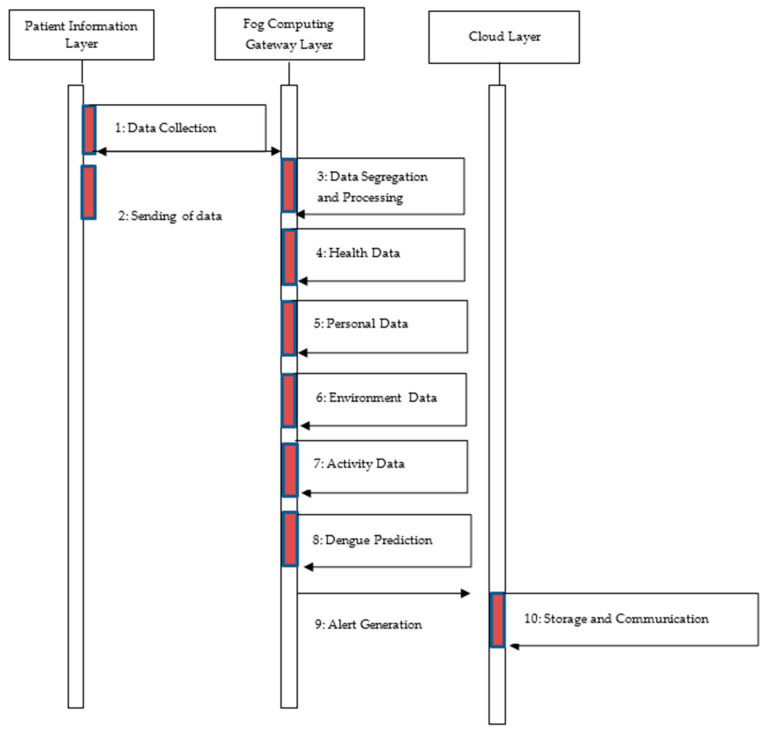
Operational sequence of the proposed monitoring system-based on fog computing.

**Figure 3:**
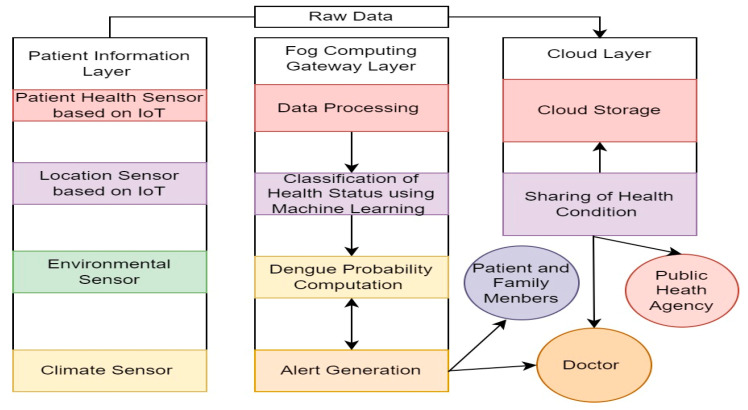
Flowchart of cloud, fog, and IoT layer to share and sense dengue data.

To evaluate and diagnose the data, the collected data are forwarded to the fog computing gateway layer. An ensemble classifier is employed in this layer to forecast dengue fever. Once dengue is anticipated, patients are notified by alert message so that preventive measures can be performed. The cloud layer’s job is to store processed data and distribute it to medical professionals, health care facilities, and patients’ families. The information is also utilized to estimate the dengue fever’s effects in a certain area. Users who will visit these locations can also receive some cautionary warnings. Figure 2 and Figure 3 illustrate the proposed DengueFog monitoring system’s flowchart. The flowchart demonstrates how each layer of the proposed monitoring system operates.

### 3.1. Patient Information Layer

The patient information layer is in charge of gathering user information based on environmental factors and sickness symptoms. The data are categorized as behavioral, personal, activity, health data. The information is gathered using a variety of wearable gadgets and sensors positioned on the subject’s body and in their environment. Additionally, using WSN technologies, the captured data are communicated in a real-time context. The following IoT sensor types are utilized to gather the required dataset of information for dengue surveillance.

- Health Dataset: Information about dengue disease symptoms can be found in the health dataset. Vomit, a fever, rashes, body aches, headaches, abdominal pain, chills, etc., are a few of the symptoms. These kinds of data are gathered for each person using a health sensor. These health sensors are Wearable Sensors like fitness trackers, smartwatches, or other health monitoring gadgets. These devices may continuously collect data on various health parameters, including body temperature, heart rate, and activity levels. For specific symptoms such as fever, body aches, and rashes, non-invasive sensors like infrared thermometers or cameras may be used to measure body temperature and detect skin conditions. Some symptoms, such as vomiting, abdominal pain, and headaches, may require self-reporting by individuals, where they input their symptoms into a health app or system.
- Environmental Dataset: Information about people’s physical surroundings is included in this dataset. In case of dengue disease, the important parameter are mosquitoes, their breeding, and locations. The other factor that can be considered for dengue disease is water sources in terms of pond, well, cooler, etc., where mosquitoes can breed. Sometimes, humidity level, temperature, rainfall parameters are also taken into consideration.
- Location Dataset: It contains the information of suspected and infected people of dengue disease. Further, the location of mosquito breeding and population is also one of the important parameters. In addition, RFID tag is used for close proximity.
- Personal Dataset: Each person’s personal information is included in the data. This dataset’s attributes include sex, address, name, qualification, occupation, etc. Therefore, each individual’s confidential information is stored in a personal dataset. Table 2 summarizes the different datasets including possible attributes and attribute types. The procedural steps of patient information layer are summarized to Algorithm 1.

**Algorithm 1:** Procedural Steps of Patient LayerStep 1:Collect the personal and behavioral data of patients.-Generate the unique id of each patient.-Enter the basic information of patient like age, gender, weight, qualification, etc.-Enter the behavioral information of patients such as occupation, working, etc. Step 2:Collect the patient physiological and health data.-Put wearable IoT devices on student body.-Collect the data related to temperature, BMI, BP, CH, etc., of the patient.-Collect health-related data of the patient like vomiting, joint pain, itching, muscle pain, skin redness, feeling of nausea, tense muscle etc. -Synchronize the structured and unstructured data of the patient.Step 3:Collect the environmental and location data.-Determine the environmental data such as humidity, rainfall, temperature, etc.-Determine the location data such as breeding side count, mosquito density, mosquito breeding sites etc.-Synchronize the heterogeneous data related to environmental and location attributes.Step 4:Data Transmission-Transfer the collected data to fog layer using wireless technologies.-Security issues should be ensured during data transfer process.

### 3.2. Fog Computing Gateway Layer

The fog computing gateway layer lies among the cloud and the patient information layer. This layer deals with processing and analyzing real-time data obtained from various IoT devices and sensors, as well as identifying patients who may have dengue infection. An alert message will be generated and sent to the appropriate patient if the patient has dengue infection (infectious, positive, and recover). Additionally, this layer is linked to the cloud layer on which the patient data are stored. Alert generation and dengue classification are the two elements that make up this layer. The procedural steps for fog computing layer are summarized in Algorithm 2.
**Algorithm 2:** Steps of fog computing LayerStep 1:Retrieve the data from repository on fog layer.Step 2:Perform the preprocessing technique on collected data.Step 3:Applied random forest classifier for dengue prediction (Algorithm 3).Step 4:Adopted Gini Index based feature selection algorithm.Step 5:Monitor the dengue affected patients and generate an alert message (Algorithm 4).Step 6:Store the data on fog computing layer for future perspective.

#### 3.2.1. Dengue Prediction

The data on dengue are divided into four classes by this module. According to Table 3, these categories are negative, infectious, positive, and recover. The patient information layer is in charge of gathering real-time, unprocessed health, personal, activity, behavioral, environmental, dengue, and IoT sensor data.

At the fog computing layer, the data are analyzed using techniques like missing value imputation. The resulting dataset is used to forecast the dengue infection and is diverse in character. The heterogeneous dengue dataset is assembled using fog node and is transformed into a special format for dengue infection prediction. Weighted random forest classifier is utilized in the fog computing layer to forecast dengue infection in patients. The proposed weighted RF classifier’s operational procedures are provided in Algorithm 3.
**Algorithm 3:** Weighted random forest algorithm for dengue predictionInput: Dengue Training Partition (P), Count of Trees (N), Features Subset—Random (FS) Output: Random Forest (RF) Tree with Dengue PredictionFor each i = 1 to N, do:Apply bootstrap algorithm on training partition (P) such as $P_{i}=\mathrm{bootstrap}\;\left(P\right).$Apply the Decision Tree (DT), ${\mathrm{DT}}_{i}=\mathrm{Random}\;\mathrm{Decision}\;\mathrm{Tree}\;\left(P,\;\mathrm{FS}\right).$Build the RF as $\;\mathrm{RF}=\mathrm{RF}\cup {\mathrm{DT}}_{i}$.End forFor each i = 1 to N, do:Calculate the weight ($w_{i}^{t}$) of ith sample using Equation (1).
(1)$$w_{i}^{t}=\frac{1}{\mathrm{OB}}\underset{j\epsilon \mathrm{OB}}{\sum }\left|X_{\mathrm{pred}}{\;}_{i,j}-X_{\mathrm{actual}}{\;}_{i,j}\right|$$End forFor each i = 1 to N, do:(2)$$\emptyset_{i}=f\left(\mathrm{AUC}{\left(w^{t}\right)}_{{\mathrm{IB}}_{i}},\mathrm{AUC}{\left(w^{t}\right)}_{{\mathrm{oB}}_{i}}\right)$$End forFor each i = 1 to, do:Calculate the weight ($w_{i}$) using Equation (3).(3)$$w_{i}=\frac{{\left(N\sum p_{i}+1\right)}^{k}}{\sum_{k=1}^{\mathrm{NT}}{\left(N-p_{i}+1\right)}^{\;k\;\prime }}$$For each i = 1 to N, do:Calculate the Final Prediction using Equation (4).(4)$$X\_{\mathrm{pred}}_{i}=\frac{1}{\mathrm{NT}}\overset{\mathrm{NT}}{\underset{j=1}{\sum }}X\_{\mathrm{pred}}_{i,j}\times w_{j}$$End forReturn RF.

#### 3.2.2. Alert Generation and Monitoring

The history and progress reports of the infected patients are periodically checked. The people who have contracted dengue are thought to be monitored at frequent intervals. In general, infectious patients are observed every three hours, and positive patients every ten. Patients recovering from dengue may take a variety of times and may vary depending on the advice of their doctor. As a result, the Probability of Dengue Index (PDI), which may be calculated using Equation (5), is used to monitor the patient.

(5) $$\mathrm{PDI}=P\left(\frac{G}{\left(H_{1}\cup H_{2}\ldots \ldots \cup H_{n}\right)}\right)$$

Equation (1) shows the probability, the current dengue class (G), and the severity of the occurrence (H_1_, H_2_…, H_n_). By following the discovery of dengue infection, a message of alert is transmitted via the fog computing layer. This alert message is delivered to the end user’s registered mobile number and consists of various PDI ranges. Patients, patients’ families, hospitals, and doctors are examples of end users. A dengue negative alert message is delivered to end users if the PDI value is normal. Users receive a warning message with information on dengue infection, if the PDI number is abnormal. These warning messages can aid medical professionals in making an early diagnosis of dengue infection. The patient can then receive the appropriate care and safety measures in response to the effects of the dengue virus. Additionally, the proposed system can reassess the dengue illness and produce alert messages. The procedure for patient monitoring is summarized in Algorithm 4.
**Algorithm 4:** Process of patient monitoringStep 1:If (Patient_Status == Dengue_Positive)Step 2:An alert message is sent patient regarding the dengue and suggest the list of paneled hospitals.Step 3:Take the appointment in the hospital and book the doctor.Step 4:Send the message to doctor regarding the patient health status.Step 5:Else if (Patient_Status == Infectious)Step 6:Inform the doctor and patient regarding the dengue infection.Step 7:An advisory is issued regarding the dengue for the patient.Step 8:Else if (Patient_Status == Recover)Step 9:Book the patient for dengue test.Step 10:Check the test results, if satisfactory, give advisory for further precaution.Step 11:Else (Patient_Status == Dengue_Negative)Step 12:No symptoms of dengue is detected in patientStep 13:End ifStep 14:Add the entry of patient into dengue dataset.

### 3.3. Cloud Layer

The processed data are stored for communication purposes in the DengueFog monitoring system using a cloud layer. Data about patients are kept on the ubiquitous cloud layer, which is accessible from anywhere at any time. The cloud database is currently not shared. It includes details about the user’s health state, personal information, social contact data, and medical history. This mode protects the privacy of the data from unauthorized access and contains highly sensitive information.

Additionally, two different sorts of authorized users can access the stored data via the cloud layer. Hospitals, doctors, or patients’ families are among these users. Affected subjects and their families can view the patient’s health report and leave comments regarding their experiences, health, and treatment. Similar patients utilize this input to guide their care in a proper and exact manner. However, in order to treat patients, hospitals and medical professionals access the patient’s data. The cloud layer’s operational procedures are displayed in Algorithm 5.
**Algorithm 5:** Process of the cloud layerStep 1:If (Patient_Id == Exist) for storing the data into cloud repositoryStep 2:Update the patient information and store it.Step 3:Else Generate the patient id. Create a new data record in the dengue dataset. Store the information of new patient in repository. End ifStep 4:To access the data from cloud repository, do followingStep 5:If (User == Doctor) -check the doctor id in database. if (doctor_id == mapped) Access the data on cloud layer Else Unauthorized user End ifStep 6:Else if (User == Patient) Check the patient id in database. if (Patient_id == mapped) Access the data on cloud layer Else Unauthorized user End ifStep 7:Else User is unauthorized, access is not granted. End if

## 4. Experimental Results

The experimental findings of the suggested DengueFog system are presented in this section. The system’s effectiveness was evaluated using the real-time dengue data set. The dataset contained the data of dengue patients of year 2018–2020 from the Delhi-NCR region. According to Table 3, there are four classes. Additionally, a WRF classifier was used in the proposed system to forecast patient dengue infection. A Windows 10 computer with an Intel Core i5 (7th generation) processor, 8 GB of RAM, and an NVIDIA GEFORCE GPU and CPU operating at 2.70 GHz was used to build the classifier. The experimental setting was further separated into three phases, including Performance Measurement, Evaluation of the Evolution of Proposed System, and Evaluation of Alert Generation.

### 4.1. Performance Management

The various performance parameters used to assess the performance of the proposed system are accuracy, F-value, specificity, precision, sensitivity, and error rate [70,71,72,73].

- Accuracy of a proposed system is defined as the ratio of accurately predicted samples to the total number of samples. For example, if there are 100 users in the dataset, 9 of them are suffering from dengue infection, but the system predicts zero dengue patient, the systems accuracy is 91/100 = 0.91%. The prediction’s accuracy is calculated by following equation:

(6) $$\;\;Accuracy=\frac{\sum TP+\sum TN}{\sum TP+\sum TN+\sum FP+\sum FN}$$

where, *TP*, *FP*, *FN*, and *TN* represent the True Positive, False Positive, False Negative, and True Negative, respectively.

- *Precision* is determined as the percentage of accurately predicted positive sample to total number of positive samples, along with *FP* samples. For example, if 9 dengue patients are predicted by system out of 100, but there are only 3 genuinely infected patients, the predicted precision is 3/9 = 0.333%. The prediction’s precision is computed by following equation:

(7) $$\;\;\;\;Precision=\frac{\sum TP}{\sum TP+\sum FP}$$

- *Recall/Sensitivity* is the ratio of correct positive samples to total positive samples; for example, if 7 dengue patients are correctly predicted by the system and 4 patients are mistakenly predicted, but in reality, there are 8 patients, the recall is 7/8 = 87.5%.

(8) $$\;\;Recall=\frac{\sum TP}{\sum TP+\sum FN}$$

- *F-Value* is defined as harmonic mean of recall and precision. It is measured as follows:

(9) $$\mathrm{F-Value}=2x\frac{recall*precision}{recall+precision}$$

- *Specificity* is the probability of a positive samples, how many patients who do not have the dengue infection and obtained negative results? It is defined using equation.

(10) $$\;\;\;Specificity=\frac{\sum TN}{\sum TN+\sum FP}$$

- *Error Rate* is the percentage of instances a decision model has categorized a sample incorrectly.

### 4.2. Evaluation of Proposed Monitoring System Based on Fog Computing

The experimental outcome of the suggested system with WRF is compared with the existing models: Decision Tree (DT), Naive Bayes (NB), Boosting, Random Forest (RF), Artificial Neural Network (ANN), and Support Vector Machine (SVM) [49,74,75,76,77]. The proposed approach and the aforementioned classifiers’ performance comparison are shown in Table 4. As can be seen, the proposed approach outperforms the traditional classifiers. Additionally, it has been shown that WRF-based health monitoring systems achieve higher precision, specificity, recall, sensitivity, accuracy, F-value rate, and lower error rates. Additionally, the DT classifier is the worst performing model. The F-value, recall, precision, accuracy, and specificity of proposed system are increased, respectively, by 35.49 %, 33.84%, 37.06%, 14.47, 12.25% from NB classifier, 39.38%, 38.85%, 40.06%, 19.64%, 13.74% from SVM classifier, 44.93%, 46.16%, 44.06%, 19.81%, 12.44% from DT classifier, 10.38%, 11.04%,10.06%, 5.34%, 4.24% from ANN classifier, 35.65%, 31.18%, 39.18%, 13.55%, 5.79% from Boosting classifier, and 10.3%, 8.46%, 12.17%, 5.37%, 1.35% from RF classifier. The error rate is decreased by 17.47%, 21.64%, 24.51%, 7.14%, 16.55% and 8.37% from NB, SVM, ANN, Boosting, and RF classifier.

**Figure 4:**
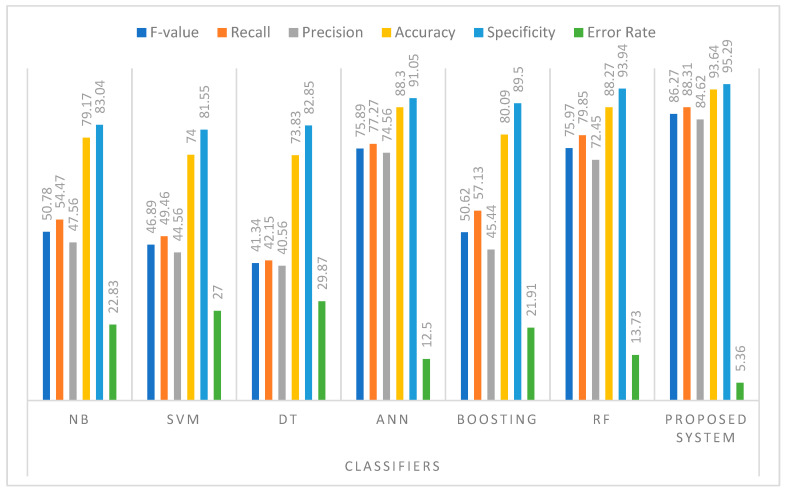
Performance comparison of proposed DengueFog system with WRF classifier and traditional classifiers.

A graphic representation of the specificity, F-value, error rate, accuracy, precision, and sensitivity rate to show efficiency of the models is shown in Figure 4. It is concluded that the proposed WRF classifier achieved a higher specificity, F-value, accuracy, precision, and sensitivity rate, and lower error rate for the dengue dataset, while the DT classifier offered the worst performance. Based on the experimental study, the following points are highlighted demonstrated in Figure 4:

- WRF classifier is used in the proposed system to forecast dengue illness, and it performs with a higher accuracy than RF. Because the effectiveness of RF classifiers relies on the quantity of the decision trees produced, RF cannot retain generality on small size hardware. In this work, the weighted technique is combined with the RF technique to solve the drawbacks of RF. The purpose of this amalgamation is to retain the generalization of RF even with fewer decision trees by leveraging the fact that sequential training creates complementary DT for training samples.
- It has been shown that NB and Boosting classifiers perform somewhat differently overall, particularly for the F-value and accuracy metrics. This is because both classifiers utilize distinct objective functions to predict dengue illness. Further, the Boosting classifier increases the time, complexity, and computation.
- The performance of the RF classifier is observed to be superior to that of the NB, Boosting, SVM, ANN, and DT classifiers. This is due to RF’s ability to be parallelized, to handle unbalanced data, its excellent high-dimensionality performance, quick prediction or fast training speed, resistance to non-linear data, moderate variance, and low bias.
- ANN depicts the complicated relationship between output and input. Therefore, it performed better than NB, Boosting, SVM, and DT classifiers.
- The DT classifier performs low compared to the other classifiers, because data are not separated linearly and they ignore some important variables in the training data.

The result of proposed system with the 10- fold cross validation technique is also presented in Table 5. Furthermore, Table 6 presents the evaluation results of the proposed system with detailed dengue class-wise performance analysis of the proposed system. The outcomes illustrate that the proposed system has a higher accuracy rate when it comes to predicting infected and recover people. The average accuracy of the proposed system with RFB is 93.64%. The RFB has an average recall of 88.31%. The higher precision rate, i.e., 84.62%, is generated by these higher accuracy and recall values, allowing the proposed system to minimize the error rate. Furthermore, the RFB generates less prediction errors due to its overall better specificity value of 95.29%. Similarly, higher F-value (86.27) and accuracy (93.64) values show that the RFB-based prediction is more accurate. Hence, it is concluded that the performance level fetched from the aforesaid parameters explains the use of RFB in the suggested system for fog-based health monitoring system.

### 4.3. Evaluation of Alert Generation

The suggested fog-based alert module offers timely information to the doctor, patient, patient’s family, and hospital about the dengue infection diagnosis. The reliability of response time and generated warnings were deemed the essential characteristics for determining the alert generation module efficiency. The proposed alert generation module was thoroughly investigated to determine its efficacy in delivering accurate and timely alerts to the doctor, patient, patient’s family, and hospital. The proposed fog-based alert module was tested on a snapdragon 636 1.8 Ghz with Octa-core processor and smartphone with 4 GB of memory and compared with EC2-based cloud instance alert generation system with no fog computing provision but with the same WRF prediction technique. Both systems were tested on the same dataset. The response time for both systems was measured from the time an event occurs to the time the warning about the event is generated and provided to the stakeholders. The alerts’ accuracy was determined in terms of the infection diagnosis procedure’s capacity to determine the genuine alarms. Table 7 compares the performance of the proposed fog-based and existing cloud-based alert generation module using various parameters such as mean absolute error, root relative squared error, maximum delay, etc., as presented in Table 7. When compared to the cloud-based system, the outcomes show that the suggested system has considerably better alert production functionality, taking approximately half the time on average to generate notifications. For accuracy, the increased recall and precision values helped to minimize the error rate and reduce the false prediction rate. The proposed system’s accessibility of resources near persons, its prevention from delay of the network communication to the cloud subsystem, and its availability of higher bandwidth and low latency in the fog-subsystem have enabled the immediate alert generation from the edge of the network of persons to reduce the error, data congestion, and data volume transmission over the network for prediction. Further, the higher accuracy, recall, and specificity values suggest that the generated warnings are reliable. The proposed alert generation module proves its utility with fewer false-positive alerts, better results, lower error rates, and improved average delay or response time.

### 4.4. Limitations and Future Directions

While the proposed system demonstrates remarkable performance, there remains room for improvement. Notably, its current scope is limited to addressing challenges specific to the dengue disease, potentially constraining its adaptability to other health-related issues. Additionally, the sensitivity of WRF model to the initial hyperparameters underscores the need for a robust optimization strategy to fine-tune these parameters effectively. To bolster the system’s overall resilience and comprehensibility, future endeavors will focus on expanding the model to incorporate features that enhance interpretability and explainability within the framework of the WRF model. This expansion aims to offer a clearer insight into the decision-making processes of the model, fostering increased trust and understanding of its outcomes across diverse user groups.

## 5. Conclusions

For monitoring and predicting the dengue infection, a monitoring system based on fog computing was proposed in this study. The proposed monitoring system is made up of three layers: the cloud, the fog computing gateway, and the patient information layer. Data on dengue cases were gathered and patient health was tracked using a variety of IoT devices and sensors. Additionally, a WRF classifier was created to predict dengue infection. To minimize the load on the cloud layer, the suggested WRF model was coupled with the fog computing gateway layer. Additionally, an alert message module that indicates the condition of dengue patients was also produced at the fog layer. By utilizing the data of 1254 dengue patients, the efficacy of the suggested DengueFog system was assessed and compared to traditional machine learning techniques. It has been found that the DengueFog system method attains a greater accuracy rate when compared to other classifiers. Additionally, the suggested system correctly sends end users alert messages. The proposed DengueFog system also notifies registered users of the dengue infection via proximity messages.

## Figures and Tables

**Table 1:** A summary of the literature referred in this study.

Author	Work Description	Data Traces
Shah et al. [42]	Addressed the problems with service quality for medical applications.	Historical patient data with real-time patient data.
Nandyala et al. [43]	Enhanced communication between hospitals and smart homes.	Designed u-health care monitoring system using cloud to fog (C2F) and IoT computing.
Costanzo et al. [44]	The main goal was to use mobile devices to monitor patients who are stationed far away. For quick patient rescue in an emergency, the suggested monitoring technique was used for interfacing by means of the first-aid software.	Wearable technology and embedded technologies-based system was devised. The overall goal of the suggested monitoring system is to suggest the appropriate course of action in cases of serious medical disorders.
Oluwagbemi et al. [45]	The suggested approach was made to diagnose and suggest treatment for the Ebola virus disease. In a survey conducted, 61% of respondents agreed that the suggested approach might suggest a course of treatment for the Ebola virus disease.	Constructed Ebola fuzzy informatics system using fuzzy logic and expert systems.
Sood et al. [46]	The proposed system was devised to track and distinguish between the numerous diseases spread by mosquitoes. The suggested system’s goal is to regulate diseases at their earliest stages. The suggested structure calculates similarity factors to distinguish between diseases.	IoT sensors, fog, and cloud computing make up the main components of the proposed health care system. The infected users are classified using the J48 decision tree classifier.
Thota et al. [47]	A security-based architecture for geographically distant health care systems was created. The tracking, identification, and security of authorization and authentication for all devices are the primary goals of the proposed design.	The suggested architecture enables asynchronous communication between cloud-based health applications and data servers.
Venckauskas et al. [48]	Datagram Transport Layer Security (DTLS) and User Datagram Protocol are replaced with the suggested protocol as a secure transport for Constrained Application Protocol (CoAP) (UDP). The experimental findings demonstrated that the suggested protocol performs better than DTLS and UDP in lossy networks and with CoAP block transfer mode.	For a fog-based eHealth architecture, a protected self-authenticable transfer protocol was proposed.
Saxena et al. [49]	A health care system was designed to manage mosquito infections at an initial stage.	The suggested solution uses wearables and IoT devices, fog computing, fuzzy k-nearest neighbor technique, and social network analysis concepts.
Ginier et al. [50]	Zika fever might be mistaken for dengue fever, though Zika infection seldom causes fever. It It has been observed that the only symptoms of a Zika infection are skin rashes and slight edema in the patient.	A discussion on the potential treatments for ZIKV infection was carried out.
Pabbaraju et al. [52]	To identify and distinguish between these viruses for the purpose of proper therapy, the RT-PCR assay was used for testing the blood of a patient. According to the findings, the RT-PCR assay is completely precise and did not exaggerate any of the several viruses examined.	Reverse Transcription—Polymerase Chain Reaction (RT-PCR) assay was used to identify the Zika, dengue, and chikungunya viruses.
Campion et al. [53]	Using data on trap counts from 2005 to 2015 and historical weather data, the authors suggested a prediction technique using the partial least squares regression technique to forecast the mosquito trap counts.	A web interface based on Google Maps was created to display information regarding the frequency of the West Nile virus, the density of mosquitoes, and the weather.
Lambert et al. [54]	An age tracking tool was developed to accurately predict the age of the mosquito. The author also argued that a crucial parameter for killing adult mosquitoes is the mosquito’s age. This objective age assessment generates a precise mosquito population.	The suggested method made use of boosted regression trees, random forests, main components regression, and neural networks with near-infrared spectroscopy, among other machine learning approaches.
Kirk et al. [55]	The system’s primary objectives are to identify environmental changes, make risk as-assessments, and provide real-time advice for mitigating mosquito illness outbreaks.	The DEAR (Detect, Evaluate, Assess and Recommend action) decision-making system was created.
Devarajan et al. [56]	A health care system was put into place dealing with the Parkinson’s disease. The suggested system examined patient voice samples to suggest best course of action.	In the suggested architecture, fog computing served as a midway layer in the end user and the cloud server. Further, the classification of Parkinson and non-Parkinson subjects was performed using the fuzzy k-nearest neighbor (FKN) classifier, case-based reasoning (CBR) classifier.
Kaur et al. [57]	The recommendations for diagnostics are provided based on the past data stored in the cloud. The judgments of how to hide the numerous patterns in the database were also aided by the suggested method.	A health monitoring system utilizing cloud concept, multiple machine learning methods, and IoT structure was described.
Parthasarathy et al. [58]	The proposed LMM system for joint inflammatory disease made use of wearable sensor devices and uric acid sensors as a component of IoT infrastructure. The suggested technique is also utilized to transform health information and identify foot motion in order to diagnose GOUT arthritis.	A leg movement monitoring (LMM) system was designed to identify the onset of disease or joint pain.
Tuli et al. [59]	The suggested model offered fog services via IoT devices and maintained medical data in accordance with user requests. Using FogBus, the implementation time, latency, power consumed, accuracy, bandwidth of network, and jitter of HealthFog are evaluated. The findings demonstrated that HealthFog offers the highest level of service quality and forecast accuracy.	A novel model called HealthFog was created for the automatic analysis of cardiac disorders. The HealthFog integrated edge computing (EC) hardware with deep learning (DL).
Priyadarshini et al. [60]	A DeepFog health care model to forecast overall wellness was developed. It used fog computing to gather patient data and deep neural networks to forecast three aspects of well-being, including stress level, hypertension attacks, and diabetes.	Fog computing and deep learning was used for constructing the model.
Jabeen et al. [61]	Recommender system was established to diagnose heart illness. The primary purpose of the suggested system is providing consumers nutrition and exercise advice.	Biosensors, IoT, prediction classifiers RF, NB, MLP, and SVM used for designing the system.
Sood et al. [62]	A cyber–physical localization (CPL) system was proposed with the fundamental goal of assessing the jeopardy of coronary heart disease, for tracking patients’ ECG readings, to inform users and specialists when readings are aberrant, and to suggest medications and preventative measures in accordance with risk category.	The proposed system is based on the concepts of cloud computation and neuro-fuzzy implication.
Gu et al. [63]	A diagnostic knowledge model (DKM) established for classifying the clinical conditions. The suggested system’s main goals are to discharge the health staff of the hefty weight of hospital duties and to offer appropriate decision-support.	The proposed system incorporated medical devices and made use of knowledge systems using the Component-Based Medical Cyber–Physical System framework (CBMCPS).
Sood et al. [64]	A health care system was introduced to identify early-stage hypertension individuals based on user health data. The suggested method continuously evaluates and keeps track of the patients’ blood pleasure.	The system uses IoT sensors, artificial neural networks, mobile devices, and cloud storage.
Lakshmanaprabu et al. [65]	A health care system was developed to categorize the various diseases according to chosen criteria. Using a precision parameter, the suggested system was assessed using several real-time hospital datasets.	The system employed an IoT structure, MapReduce, the enhanced dragonfly algorithm, and RF classifier.
Anand et al. [66]	A hybrid framework was suggested to categorize the hepatic syndrome. The suggested system’s performance was assessed, and the findings proved that it outperforms as compared to existing systems classification accuracy.	The techniques used are updated particle swarm optimization, updated artificial neural network, the SPARK tool.
Sood et al. [67]	A diagnostic system suggested that incorporates social network analysis (SAS) in cloud subsystem to offer a GPS-based worldwide risk assessment of dengue infection on Google Maps for preventing the spread of the infection. The effectiveness of the suggested system’s diagnosis, warning production, and risk assessment based on GPS capability was acknowledged using various statistical measurements and experimental methodologies.	A system with NB network and fog computing suggested and used Google Maps, GPS, SAS.
Sood et al. [68]	An IoT-based fog-cloud diagnosed system for controlling and detecting dengue infection in 2021. To analyze the influence of the proposed system, the investigational findings were assessed using a numeral of analytical constraints.	The proposed system uses SVM, Google Maps, and temporal network analysis (TNA).
Suggala et al. [69]	A novel dengue prediction method using fog computing introduced. The dengue infected was detected by checking the similarity factors between the disease and the users. Finally, at the cloud layer, an innovative Temporal Social Network Analysis (TSNA) was designed to evaluate the risk of disease outbreak, analyze sick users, and direct an awareness text to initiate preventive steps.	The proposed method uses cloud concept and temporal social network analysis (TSNA).

**Table 2:** A summary of the information of different datasets collected in this study.

Dataset	Symptoms/Attributes	Attribute Type	Attribute Sub Type
Health-Related Data	Fever, Vomit, Severe Body ache, Severe Headache, Nausea, Abdomen Pain, Joint Pain, Pain Behind Eye, Muscle Pain, Skin Rashes, Soft Bleeding, Red Eye, Appetite Loss, Yellow Skin	Qualitative attributes	Binary Nominal
Environmental related data	Humidity, Temperature, Rainfall Parameters, Carbon Dioxide Level	Quantitative and Qualitative attributes	Numeric and Nominal
Location related data	Breeding Side Count, Mosquito Density, Mosquito Breeding Sites, Individual Count on Site	Quantitative and Qualitative attributes	Numeric and Nominal
Personal data	Unique ID Number, Name, Sex, Qualification, Occupation, Phone Number, Workplace Address, Home Address	Quantitative and Qualitative attributes	Numeric, Binary Nominal, Nominal

**Table 3:** Dengue class classifications and description.

Dengue Class	Description
Negative	Patient exhibits no indications of illness
Infectious	Patient has red eyes, high fever, abdominal pain, bleeding disorder, low level of immunity and muscle pain
Positive	Patient has fatigue along with headache and skin rashes
Recover	Patient has no more infection

**Table 4:** Performance comparison of proposed DengueFog system with WRF classifier and traditional models.

Performance Measurement	Classifiers						
	NB	SVM	DT	ANN	Boosting	RF	Proposed System
F-value	50.78	46.89	41.34	75.89	50.62	75.97	86.27
Recall	54.47	49.46	42.15	77.27	57.13	79.85	88.31
Precision	47.56	44.56	40.56	74.56	45.44	72.45	84.62
Accuracy	79.17	74.00	73.83	88.30	80.09	88.27	93.64
Specificity	83.04	81.55	82.85	91.05	89.50	93.94	95.29
Error Rate	22.83	27.00	29.87	12.5	21.91	13.73	05.36

**Table 5:** 10-Fold classification results of proposed system.

Sr. No.	Precision	Recall	F-Value	Specificity
1	82.8856	82.1866	82.5346	98.8906
2	81.6784	81.8796	81.7789	99.5467
3	82.9099	79.4185	81.1267	98.3456
4	82.1519	83.6777	82.9078	98.2733
5	82.4562	81.1258	81.7856	99.6789
6	82.0067	85.8945	83.9056	99.8902
7	82.1224	82.0996	82.1110	99.6783
8	82.8112	84.9677	83.8756	98.0045
9	83.0789	79.4702	81.2345	99.5756
10	83.0707	83.5642	83.3167	98.8172

**Table 6:** Detailed dengue class-wise performance of WRFB.

Dengue Class	Precision	Recall	F-Value	Accuracy	Specificity
Negative	83.93	87.17	85.52	89.65	93.55
Infectious	85.76	87.74	86.74	95.96	97.67
Positive	88.82	83.07	85.85	93.08	94.87
Recover	79.97	95.29	86.96	95.87	95.08

**Table 7:** Alert Generation Module Comparison.

Parameters	Proposed System	Cloud-Based Health Monitoring System
Precision	84.62	76.55
Recall	88.31	80.13
Specificity	95.29	84.32
False Positive Rate	7.65	22.86
Mean absolute error	5.36	15.08
Average Delay	6.32 s	11.33 s
Maximum Delay	9.45 s	19.56 s
Minimum Delay	2.54 s	6.12 s
Delay in standard deviation	2.61	1.05 s
Relative absolute error	8.45	16.76
Root relative square error	36.89	45.02
Root average square error	3.80	9.66
Coverage	94.13%	83.78%

## Data Availability

Data sharing not applicable to this article as no datasets were generated or analyzed during the current study.
